# An Enhanced Map-Matching Algorithm for Real-Time Position Accuracy Improvement with a Low-Cost GPS Receiver

**DOI:** 10.3390/s18113836

**Published:** 2018-11-08

**Authors:** Jeong Min Kang, Han Sol Kim, Jin Bae Park, Yoon Ho Choi

**Affiliations:** 1 Department of Electrical and Electronic Engineering, Yonsei University, Seoul 03722, Korea; kangjm@yonsei.ac.kr (J.M.K.); solsol@yonsei.ac.kr (H.S.K.); 2 Department of Electronic Engineering, Kyonggi University, Suwon 16227, Kyonggi-Do, Korea; yhchoi@kyonggi.ac.kr

**Keywords:** low-cost GPS receiver, position estimation, ICP algorithm, map-matching, embedded system

## Abstract

This paper proposes a real-time position accuracy improvement method for a low-cost global positioning system (GPS), which uses geographic data for forming a digital road database in the digital map information. We link the vehicle’s location to the position on the digital map using the map-matching algorithm to improve the position accuracy. In the proposed method, we can distinguish the vehicle direction on the road and enhance the horizontal accuracy using the geographic data composed of the vector point set of the digital map. We use the iterative closest point (ICP) algorithm that calculates the rotation matrix and the translation vector to compensate for the disparity between the GPS and the digital map information. We also use the least squares method to correct the error caused by the rotation of the ICP algorithm and link on the digital map to eliminate the residual disparity. Finally, we implement the proposed method in real time with a low-cost embedded system and demonstrate the effectiveness of the proposed method through various experiments.

## 1. Introduction

The development of the information technology(IT) industry has recently been applied to various fields, and has especially been influencing the development of the unmanned industry. The autonomous driving technology of unmanned vehicles is the core of this industry, and the development is accelerating existing automobile manufacturers. Various IT companies have also begun to pay attention to the smart car market. Unmanned ground vehicles for autonomous driving must judge and drive to the given destination. Four representative technologies are considered when performing a complete unmanned autonomous drive to a destination: the position estimation technology that can obtain the current position of the vehicle, the environment perception technology that can recognize the surrounding environmental information by utilizing various sensors, the obstacle avoiding technology that can move without collision to an object to the given destination, and the path planning technology that generates an optimal path to a destination in a given environment. The position estimation for finding the current position of a vehicle is particularly a basis for the autonomous driving system [1,2,3,4].

The majority of the contemporary position estimation technologies are generally based on the global navigation satellite system (GNSS), such as the global positioning system (GPS). Many studies and advances in technology have improved the accuracy of satellite-based location equipment. A single-point position accuracy of GNSS is 10 m in open sky environment with many satellites and no degraded signal [5]. However, the performance of single GNSS cannot guarantee an accurate and continuous positioning in signal-degraded environments. In order to provide a remedy for the shortcomings of the single GNSS performance, a possible approach is the use of multi-constellation. The methods of the determination of the intersystem time offsets, which is characterized by a relative bias and drift between GPS and Galileo time scales, are proposed [6,7]. In particular, integrity monitoring has a great importance in safety critical operation, and the research for the performance assessment of the capabilities of integrity algorithms defined as receiver autonomous integrity monitoring (RAIM) are proposed [8,9]. In addition, the studies on improving the position accuracy, such as using the reference station that knows an accurate position, using additional sensors, and utilizing a digital map, have been actively performed.

An easy approach to compensate for these shortcomings is to use differential GPS (DGPS). DGPS is a method of inverting the errors contained in each satellite signal at the reference station, where the exact position is known, and improving the accuracy using a separate communication network. Carrier phase DGPS (CDGPS) is the augmentation system for carrier-phase measurements. This measurement obtains much less noise; however, its disadvantage is that the reliability problem occurs because of an integer ambiguity. A real-time kinematic-GPS method can be used for the spatial decorrelation in CDGPS, which improves the accuracy by efficiently calculating an integer ambiguity [10,11,12].

Other methods utilize additional equipment and sensors to perform the position estimation. Dead-reckoning using extra sensors, such as Inertial Measurement Unit (IMU), and speed measurements have been used to enhance the positioning precision and availability. In addition, an integrated navigation method combining GPS and IMU, a method by detecting lane using image information and lateral distance information of lane [13], matching the three-dimensional (3D) point cloud information generated during driving through Light Detection And Ranging(LIDAR) [14], and matching the road geometry information based on vision and radar sensors [15] have been proposed. However, these methods require separate communication links or additional infrastructure and sensors; hence, drawbacks of demanding a large amount of calculation and costs have been considered.

Another method for enhancing the position accuracy is the map-matching algorithm using a precise digital map. Most map-matching algorithms are tailored toward mapping the current position onto a vector representation of a road network to the closest node or the shape point, which is known as the point-to-point method [16]. The point-to-curve matching method also exists, which matches a location point to a curve closest to the network [17]. A weighting method of the link has been proposed to further improve the accuracy of the map-matching algorithm [18]. These methods are easy to perform, but the amount of computation and the position error increase as the link becomes more complex. Another geometric approach is to compare the trajectory of a vehicle with the road of the digital map. A method employing both distance and topology with low sampling rate, and considering the mutual influences of the GPS points in a trajectory, is proposed [19]. This method achieves better accuracy at a higher computational cost. A method for finding a moderate sampling rate utilizing user traveling behavior based on the observation that all possible segments on the map is proposed [20]. These methods have outperformed for single trajectory, but they are insufficient for real-time application with low sampling processes. However, a probabilistic map-matching method that can recover from mis-matches quickly [21], and an advanced map-matching algorithm that applies a fuzzy logic [22] is proposed. However, these algorithms still perform poorly when the sampling rate is low. A commonly used criterion for evaluating these trajectory map-matching methods is correct segment identification [23]. In order to determine the matching accuracy, the horizontal distance from the center of road is calculated. The horizontal errors in previous research are 9 m and 5.5 m [19,22]. However, these approaches cannot present the position error of each point. The methods of the map-matching algorithm based on additional sensors have also been presented. A localization method using a monocular camera and a 3D compact semantic map [24] and a map-based localization method using the curb extracted by 3D LIDAR [25] are presented. However, these methods have disadvantages of requiring additional sensors and high computational complexity. Therefore, a method based on the iterative closest point (ICP) algorithm using only the GPS and digital map information has been proposed, which does not require additional sensors [26]. This method matches the disparity between the trajectory of the GPS and the digital map information, and is considered only in a single direction using the center of the road.

In this paper, we propose a method for enhancing the position accuracy of a low-cost GPS receiver based on map data without using any additional sensors. The points extracted from the roads prior to operating the algorithm are utilized as map data. They are stored in the form of vector data, including the position and direction information. We refer to these points as the vector points in the discussion that follows. A source point set is updated at each sampling time and contains the vector points of the vehicle trajectory acquired by the GPS. A reference point set is created from the vector points in the map point set, whose direction is parallel to those of the source point set at each sampling time. In this configuration, the ICP algorithm is employed to compute the transformation between the reference point set and the source point set. The vehicle trajectory is corrected after computing these errors. The position information is further corrected using the least squares method to compensate for the error caused by the rotation of the ICP algorithm and linked on the digital map to eliminate the residual disparity. Finally, we implement the proposed method in real time with a low-cost embedded system and demonstrate the effectiveness of the proposed method through various experiments.

## 2. Preliminaries

### 2.1. Specifications

The map-matching algorithm proposed herein can enhance the position accuracy. The characteristics of the proposed method are as follows:

- The performance of a low-cost GPS receiver is enhanced based on a GPS and the digital map information without using any additional sensors.
- The proposed method is simple. The necessary information includes the *x*-*y* coordinates, and heading. By ignoring the map data with no relation to the trajectory of a vehicle, computational efficiency can be enhanced. Thus, it is not limited by the computing power of a processor.
- The performance of the proposed method is verified through real-time experiments on a low-cost MCU-based embedded board.

### 2.2. Description of the Vector Point Sets

Under the assumption that a vehicle maneuvers along roads, the objective of the proposed method is to enhance the accuracy of low-cost GPS receivers by matching the acquired GPS trajectory with a pre-constructed road map database. These can be extracted by hand or automatically using image processing methods [27]. Once extracted, they are stored in the form of vector points, including the position and direction information. Herein, the *k*-th vector point $v_{k}$ consists of the position and the direction, which has the following form:

(1) $$v_{k}=\left(p_{k},\theta_{k}\right),$$

where $p_{k}=\left(x,y\right)$; *x*, and *y* represent the latitude and longitude coordinates, respectively; and $\theta_{k}$ is the heading.

A vector point consists of the position and direction. The position is represented in the universal transverse mercator coordinate system. The heading is acquired by GPS information. In the case of a map database, it is obtained manually from the bearing on the map. The ${\bar{\theta }}_{k}$ has one of the following four values:

(2) $$\begin{array}{c} {\bar{\theta }}_{k}=\left[\begin{array}{cc} East, & \;\;\;45^{\circ }\leq \theta_{k}<135^{\circ },\\ South, & \;\;\;135^{\circ }\leq \theta_{k}<225^{\circ },\\ West, & \;\;\;225^{\circ }\leq \theta_{k}<315^{\circ },\\ North, & \;\;\;315^{\circ }\leq \theta_{k}\;or\;\theta_{k}<45^{\circ }. \end{array}\right. \end{array}$$

The proposed method utilizes the following vector point sets:

1. Assuming that a vehicle is traveling along the center of road, the map point set (MPS) is built prior to execution and composed of the vector points extracted from the center of roads.
2. The source point set (SPS) is updated from a GPS receiver at each sampling time and contains the vector points of the vehicle trajectory.
3. The reference point set (RPS) is created from the selected vector points in the MPS that a vehicle is expected to pass.

**Figure 1:**
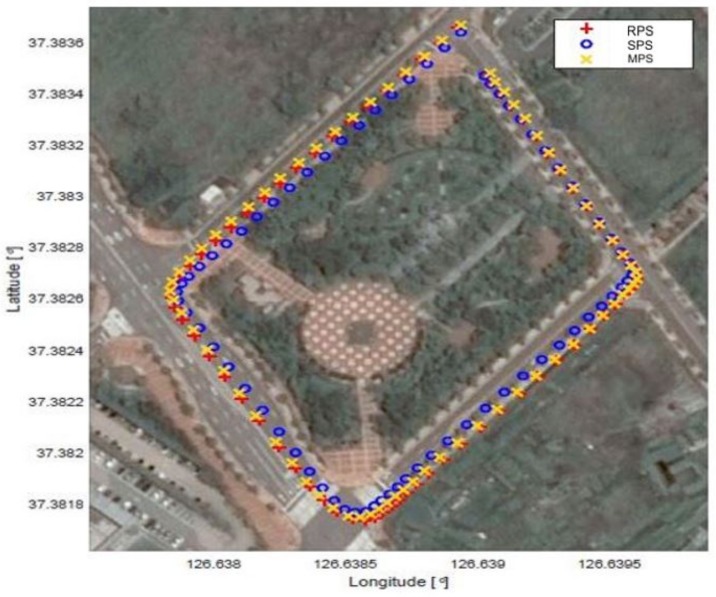
Trajectories of a vehicle.

We present some examples of the MPS, SPS, and RPS as shown in Figure 1 to clarify the definitions of these vector point sets. In these examples, the SPS contains only the vector points, whose heading direction is east or south. Therefore, it is reasonable for the RPS to be constructed from the MPS vector points, whose heading direction is east or south. This example demonstrates why the RPS is introduced in the proposed algorithm.

In short, the MPS is the pre-built fixed road map database, and the SPS and RPS are updated at each sampling time from the GPS receiver and the MPS, respectively. For simplicity, we will denote the MPS, SPS, and RPS as M, $S_{k}$, and $R_{k}$, respectively, where k denotes the *k*-th sampling time. The MPS M is further divided according to the direction, that is, $M=M^{E}\cup M^{S}\cup M^{W}\cup M^{N}$, where each set only contains the vector points, whose heading direction is north, east, west, or south, respectively. Moreover, the *i*-th vector point $v_{i}$ of any vector point set χ is represented as $v_{i}=\left(p_{i},{\bar{\theta }}_{i}\right)=\left(\chi^{P}\left(i\right),\chi^{H}\left(i\right)\right)$, where $\chi^{P}\left(i\right)$ and $\chi^{H}\left(i\right)$ are the position and heading of the *i*-th vector point of χ, respectively.

### 2.3. ICP-Based Map-Matching Method

**Figure 2:**
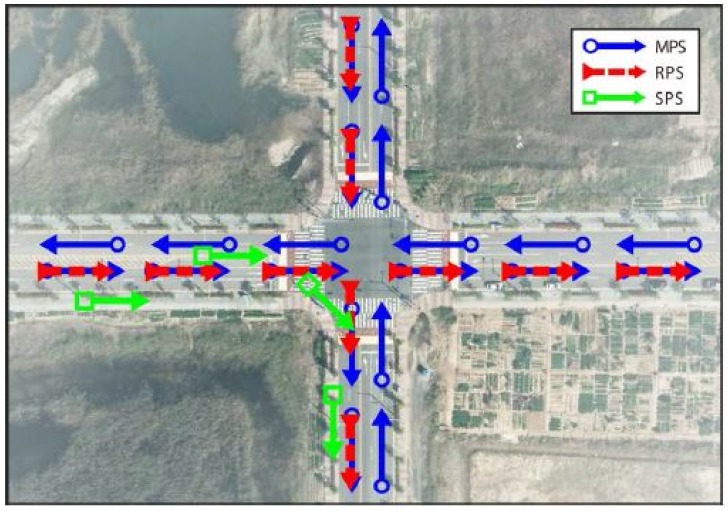
Examples of the RPS, SPS, and MPS.

Figure 2 shows the examples of the RPS, SPS, and MPS. The trajectories can be matched by rotating and translating one to fit the other. We can observe such trajectories in many scenarios. In this case, we can compute the rotational and translational errors between the trajectories using the ICP algorithm, which is widely used in field image processing. The ICP algorithm, which is a matching algorithm proposed by Besl and McKay [28], can be started with two point sets. Similarly, the GPS trajectory is composed of the vector points describing the trajectory of a vehicle for a certain period of time. The reference point set also consists of the vector points. In this configuration, the ICP algorithm is used for matching $p_{k}$ among these vector point sets. Given these sets, the ICP algorithm initiates a rotation matrix and a translation vector and finds a transformation matrix that contains a rotation matrix and a translation vector by minimizing the error function. The error function *J* is defined as follows:

(3) $$E=\arg\min_{R,t}\left(\sum_{\begin{array}{c} i=1 \end{array}}^{N_{p}}∥m_{i}-Rp_{i}-t{∥}^{2}\right),$$

where $m_{i}$ and $p_{i}$ are the corresponding points, and $N_{p}$ is the total number of points. The optimization problem can be solved by the iterative approach. A transformation matrix is obtained after the iteration procedure. We obtain the corrected GPS trajectory as shown in Figure 2 by correcting the rotational and translational errors. As can be seen, a low-cost GPS receiver provides a sufficiently reasonable performance when the ICP algorithm is employed. Thus, this study proposes a method to improve the accuracy of a low-cost GPS receiver based on the ICP algorithm.

## 3. Enhanced Map-Matching Algorithm for Real-Time Position Accuracy Improvement

**Figure 3:**
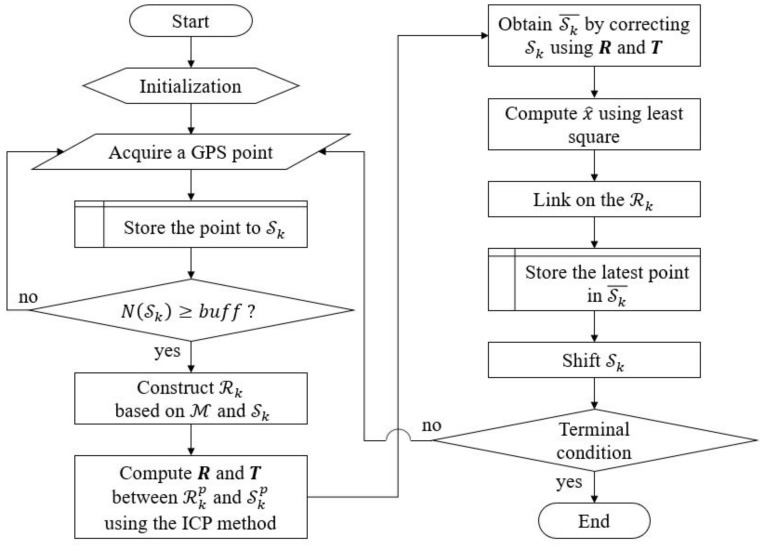
Flowchart of the proposed method.

The main idea of the proposed method is to match the SPS $S_{k}^{P}$ with the RPS $R_{k}^{P}$, where $k\in Z_{>0}$ denotes a sampling time. Figure 3 shows a flowchart of the proposed method, which could be divided into four main steps as follows:

step 1. Loading of the digital databases,
step 2. Calculation of the disparity information,
step 3. Calibration of the rotational error using the least squares method,
step 4. Location of the vehicle position on the map.

### 3.1. Step 1: Loading of the Digital Databases

**Figure 4:**
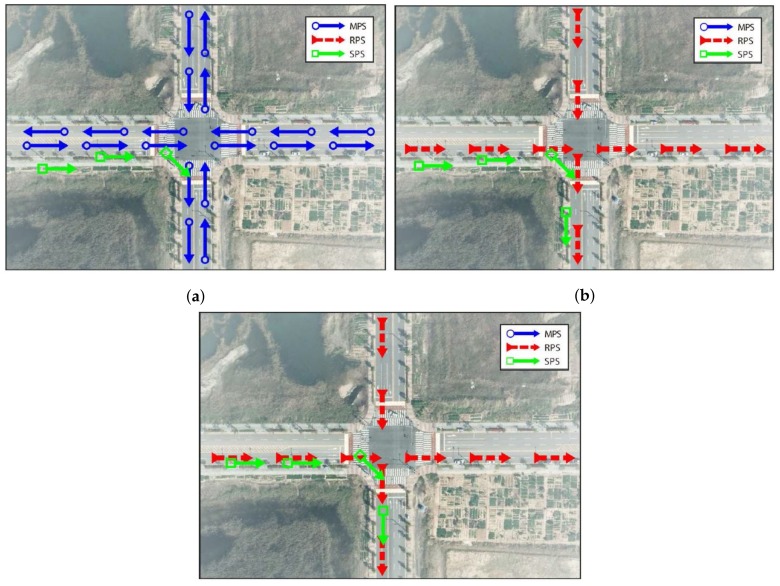
Examples of the map-matching process: (**a**) M and $S_{k}$ at k=3; (**b**) $R_{k}$ and $S_{k}$ at k=4; and (**c**) $R_{k}$ and ${\bar{S}}_{k}$ at k=4.

In the initialization stage, the MPS M is loaded from the memory, and the variables required in the proposed method are declared and initialized. After initialization, the current position and heading of a vehicle are acquired from the GPS receiver through homegrown software and stored in the SPS $S_{k}$ at each sampling time k. The method skips to the GPS acquisition stage of the (*k* + 1)th sampling time before $N(S_{k})>\mathrm{buff}$, where $N(S_{k})$ is the number of vector points stored in the SPS $S_{k}$, and buff is the predefined scalar value. Figure 4 shows the examples of map-matching process. At *k* = 3, examples of M and $S_{3}$ are shown in Figure 4a to clarify the procedure.

The position correction method begins when $N(S_{k})>\mathrm{buff}$ is satisfied for a certain k. The first step is to create the RPS $R_{k}$ based on the MPS M and the SPS $S_{k}^{H}\left(i\right)$ for i=1,2,…,buff. Algorithm 1 summarizes this process. We anticipate that a vehicle travels along a road, whose headings are parallel to that of a vehicle. Therefore, all of the MPS M(i), whose headings are equal to the SPS $S_{k}^{H}\left(j\right)$ for all possible combinations of *i* and *j*, are chosen as the vector points of the RPS $R_{k}$. Constructing the RPS $R_{k}$ is important for two reasons. First, the computational efficiency can be enhanced by ignoring the vector points of the MPS M with no relation to the trajectory of a vehicle. Second, the horizontal accuracy is enhanced by considering the direction of a road. Figure 4b shows examples of $R_{4}$ and $S_{4}$ for buff=4. In this example, $S_{4}$ contains the vector points, whose heading directions are east and south. Thus, the method ignores the roads heading in directions to the west and north. $R_{4}$ consists of M(i) whose $M^{H}\left(i\right)=East\;or\;South$ (i.e., $R_{4}=M^{E}\cup M^{S}$).

**Algorithm 1** Algorithm for constructing the RPS   **function** Construction ($S_{k}^{H}$)      **Initialize:**
$R_{k}$ ← empty set      **for** i ← 1 to *buff*
**do**            **if**
$S_{k}^{H}\left(i\right)$ is North **then**                  $R_{k}$ = $R_{k}$ ∪ $M^{N}$                  $R_{k}$ = $R_{k}$ − $M^{S}$            **else if**
$S_{k}^{H}\left(i\right)$ is South **then**                  $R_{k}$ = $R_{k}$ ∪ $M^{S}$                  $R_{k}$ = $R_{k}$ − $M^{N}$            **end if**            **if**
$S_{k}^{H}\left(i\right)$ is East **then**                  $R_{k}$ = $R_{k}$ ∪ $M^{E}$                  $R_{k}$ = $R_{k}$ − $M^{W}$            **else if**
$S_{k}^{H}\left(i\right)$ is West **then**                  $R_{k}$ = $R_{k}$ ∪ $M^{W}$                  $R_{k}$ = $R_{k}$ − $M^{E}$            **end if**      **end for**      **Return**
$R_{k}$   **end function**


### 3.2. Step 2: Calculation of the Disparity Information

After constructing $R_{k}$, the ICP algorithm is employed to compute the rotational and translational errors between $R_{k}^{P}$ and $S_{k}^{P}$. The ICP algorithm outputs the rotation matrix and the translation vector in the following forms:

(4) $$R=\left(\begin{array}{cc} cos\theta_{E} & -sin\theta_{E}\\ sin\theta_{E} & cos\theta_{E} \end{array}\right),T=\left(\begin{array}{c} x_{E}\\ y_{E} \end{array}\right),$$

where $\theta_{E}$ is the rotational error between two vector point sets, and $x_{E}$ and $y_{E}$ are the translational errors of each axis.

The estimation value of reference can be derived by calculating the transformation to the $S_{k}^{P}$. Using Label (4), $S_{k}$ is corrected as follows:

(5) $${\bar{S}}_{k}^{P}\left(i\right)=RS_{k}^{P}\left(i\right)+T,for\;i=1,2,\ldots ,\mathrm{buff},$$

where ${\bar{S}}_{k}^{P}\left(i\right)$ is the corrected trajectory of a vehicle.

Figure 4c shows $R_{4}$ and ${\bar{S}}_{4}$, from which we can conclude that the horizontal position error is minimized.

### 3.3. Step 3: Calibration of Rotational Error Using the Least Squares Method

In the proposed method, the ICP is performed by maintaining a constant number of buffer and shifting the information from which the new GPS information is obtained. The ICP algorithm returns the one transformation between $R_{k}^{P}$ and $S_{k}^{P}$. This transformation does not sufficiently reflect the change value between the trajectory and the GPS information, which are newly obtained. The ICP algorithm is more sensitive to rotation than translation [29]; hence, we use the least squares method to calibrate the rotational error. From ${\bar{S}}_{k}^{P}$, which is the corrected trajectory of a vehicle, the output equation is as follows [30]:

(6) $$z_{k}=H_{k}x_{k}+w_{k},$$

where $z_{k}$ is the latest point of ${\bar{S}}_{k}^{P}$, $H_{k}$ is the output matrix, $x_{k}$ is the state vector, and $w_{k}$ is the measurement noise. We can then compute the state estimation ${\hat{x}}_{k}$ that minimizes the cost function *J*, where *J* is given as follows:

(7) $$J={\left(z_{k}-H_{k}{\hat{x}}_{k}\right)}^{T}\left(z_{k}-H_{k}{\hat{x}}_{k}\right)=z_{k}^{T}z_{k}-{\hat{x}}_{k}^{T}H_{k}^{T}z_{k}-z_{k}^{T}H_{k}{\hat{x}}_{k}+{\hat{x}}_{k}^{T}H_{k}^{T}H_{k}{\hat{x}}_{k}.$$

We compute the partial derivative as equal to zero to minimize *J* with respect to ${\hat{x}}_{k}$:

(8) $$\frac{\partial J}{\partial {\hat{x}}_{k}}=-z_{k}^{T}H_{k}-z_{k}^{T}H_{k}+2{\hat{x}}_{k}^{T}H_{k}^{T}H_{k}=0.$$

Solving (8) for ${\hat{x}}_{k}$, the state estimation is as follows:

(9) $${\hat{x}}_{k}={\left(H_{k}^{T}H_{k}\right)}^{-1}H_{k}^{T}z_{k}.$$

In this procedure, the state estimation ${\hat{x}}_{k}$ implies the current position of a vehicle.

### 3.4. Step 4: Location of the Vehicle Position on the Map

The state estimation $\hat{x}$ is linked to $R_{k}$ to eliminate the residual disparity. The projection method uses the vector projection theorem, which is from the current state estimation and the two points near the estimation point. The lateral error is reduced, and the vehicle position is located on the road of the digital map, implying that the accuracy of the low-cost GPS receiver has been enhanced.

Finally, the FIFO buffer, $S_{k}$, is shifted, and the method waits until a new GPS point is received. The memory requirement does not increase as $N(S_{k})$ is confined to buff, even if the proposed method operates for a long time. The execution time is mainly affected by $N(R_{k})$ and $N(S_{k})$. $N(S_{k})$ plays an important role in the proposed method; hence, larger values of $N(S_{k})$ not only enhance the performance of the method, but also increase the computational load. Thus, this parameter should be chosen according to the desired performance of the proposed method. In addition, the computational burden increases with the map size, which can be solved by confining the region of $R_{k}$ based on the current position of a vehicle.

## 4. Experimental Setup and Results

### 4.1. Hardware Implementation

**Figure 5:**
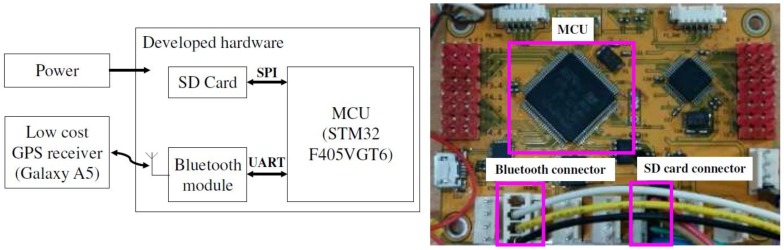
System block diagram and photograph of the developed embedded system.

The proposed method is computationally efficient; hence, it can be implemented using a low-cost embedded system. We developed the embedded system as shown in Figure 5, in order to demonstrate the efficiency of the proposed method. This system consisted of a low-cost processor, a secure digital (SD) card slot, and a Bluetooth module. The main processor was an STM32F405VGT6 (ST Microelectronics, Inc., Geneva, Switzerland) [31], which operates at a frequency of 168 MHz, and has a static RAM of 192 Kbytes. The SD card was used to store the experimental results and M. The Bluetooth module was employed to receive GPS points from a smartphone. A Galaxy A5 (Samsung Electronics, Inc., Suwon, Korea) smartphone, which provides a trajectory with a horizontal accuracy of up to 10 m, was connected to our system through the Bluetooth connectivity. The sampling time of the proposed method is set to 1 Hz, that is, one GPS point per second is sent to the embedded board.

### 4.2. Experimental Setup

We employed a high-end GPS/INS device (NovAtel, Inc., Calgary, Canada) composed of UIMU-H58 [32], SPAN-SE-D-GENERIC [33], and GPS-703-GGG [34] to obtain the actual trajectory of a vehicle. In this configuration, the device provides the position of a vehicle with an accuracy of 0.4 m; thus, it is reasonable to assume that the position acquired by the device is the ground truth.

**Figure 6:**
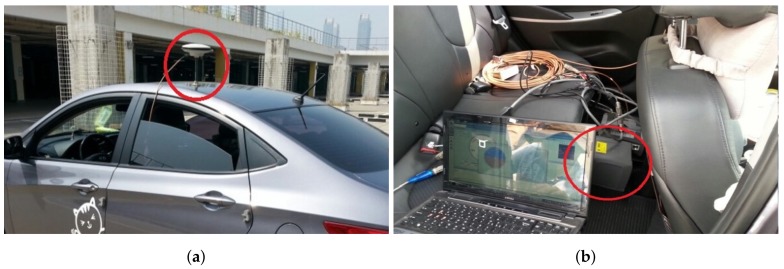
Setting of the experimental devices: (**a**) an antenna on the vehicle and (**b**) IMU in the vehicles.

The main purpose of our experiments was to verify that a trajectory acquired by a low-cost GPS receiver is effectively corrected by the proposed method. We also showed that implementing the proposed method in real time was possible using the low-cost embedded system. The GPS/INS device, a smartphone, and an embedded board were positioned on the vehicle as shown in Figure 6.

**Figure 7:**
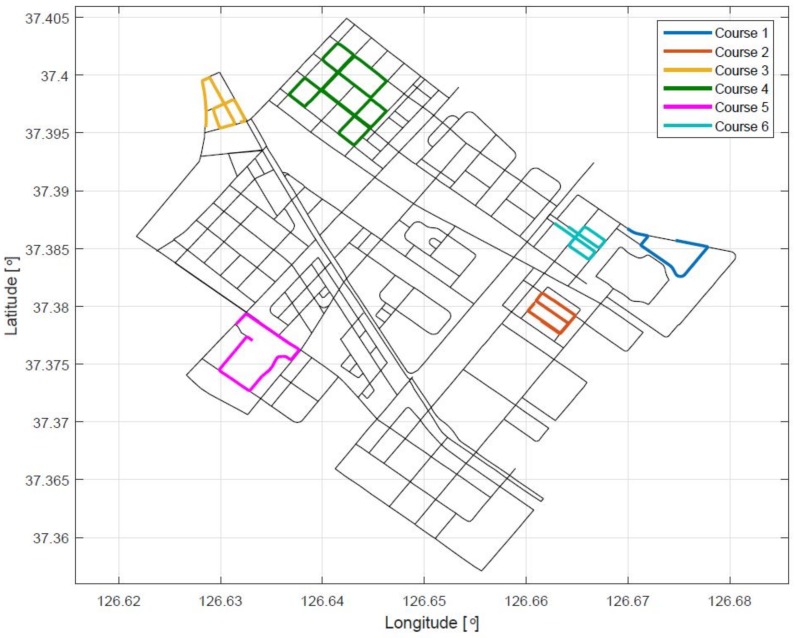
Overall map comprising six courses.

We performed experiments for the six courses shown in Figure 7. Table 1 lists the length of the paths and the number of the GPS points for each experiment.

### 4.3. Experimental Results

**Figure 8:**
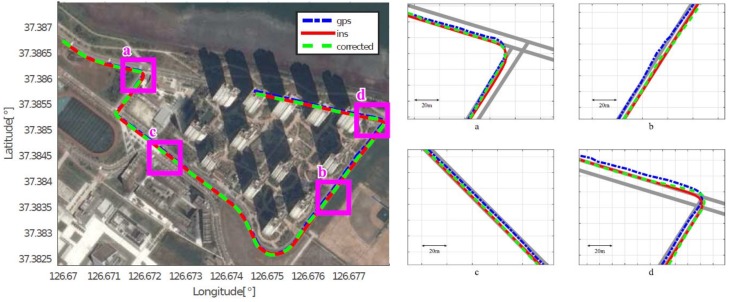
Experimental results for Course 1.

**Figure 9:**
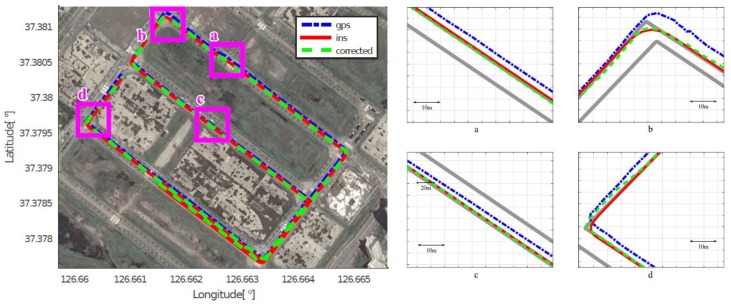
Experimental results for Course 2.

**Figure 10:**
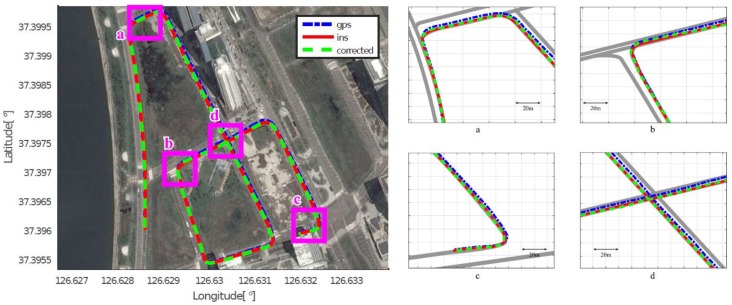
Experimental results for Course 3.

**Figure 11:**
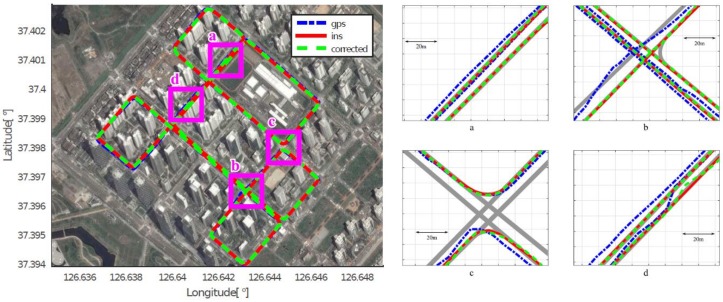
Experimental results for Course 4.

**Figure 12:**
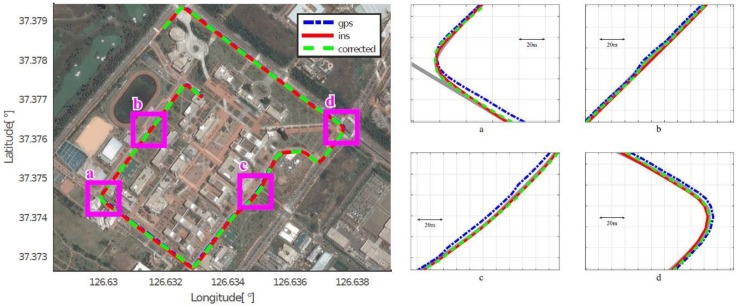
Experimental results for Course 5.

**Figure 13:**
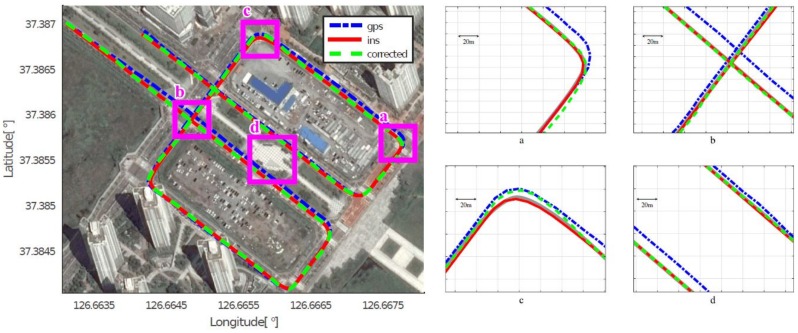
Experimental results for Course 6.

As mentioned in Section 4.2, we assumed that the position acquired by the high-end GPS/INS device was the ground truth, and calculated the position errors of the low-cost GPS and the proposed method, respectively. Figure 8, Figure 9, Figure 10, Figure 11, Figure 12 and Figure 13 show the six courses on real routes containing curved roads and intersections, including the low-cost GPS measurements, the final corrected trajectories through the proposed method, and the ground truth. Each figure contained the overall trajectories and four magnified subsections.

These figures show that, for all cases, including straight lanes, curved lanes, and intersections, the corrected trajectories were located much closer to the ground truth compared to those originally given by the low-cost GPS receiver. Figure 8c and Figure 11b,c also depict that the trajectories acquired by the low-cost GPS receiver were located on the opposite side of the roads; however, the proposed method determined which side of the roads a vehicle was traveling on. If the vehicle traveled along the center of road, the trajectory from the high-end GPS/INS device would be on the RPS. Therefore, the horizontal error between the points acquired by the proposed method and the high-end GPS/INS device was close to zero. However, the corrected trajectory sometimes cannot follow the curved lanes as the accumulated GPS points are used to correct the trajectory (e.g., Figure 13c).

**Figure 14:**
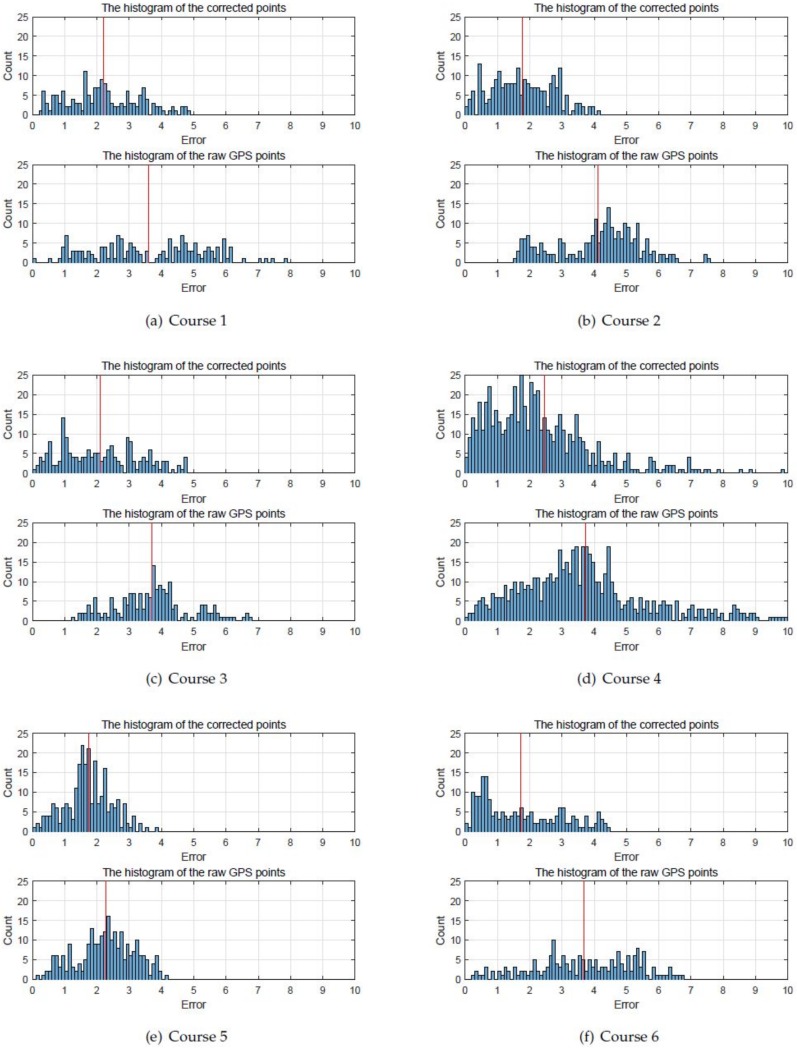
Error histograms. The blue bars represent the error distributions, while the red line marks the average value of each error distribution.

The error distributions of the proposed method and the raw GPS points for the six courses were summarized in Figure 14 to quantitatively evaluate the performance of the proposed method. In this figure, the horizontal axis represents the error that is calculated in meters. The blue graph denotes the error distribution that explains how many times each error occurred, while the red line shows the mean of each error distribution. The proposed method generated much smaller distance error than the raw GPS. The mean and the standard deviation of these error distributions were listed in Table 2 for further clarification. The columns labeled ‘Avg. (GPS)’ and ‘Avg. (Prop.)’ represent the root mean square error between the position acquired by the GPS/INS device and that of the low-cost GPS receiver and the proposed method, respectively. The ‘Std.’ columns list the standard deviations of each error distribution. The errors in the ‘Avg. (Prop.)’ column are mainly from the vertical errors because the proposed method only improved the horizontal accuracy. However, the merit of the proposed method remains in that it reduces the horizontal error. The ‘Improv.’ represents the improvement rate which is obtained by dividing the difference between ‘Avg. (GPS)’ and ‘Avg. (Prop.)’ by ‘Avg. (GPS)’, from which we can conclude that the proposed method improves the accuracy of the low-cost GPS receiver by up to 62.2927%.

We ignored the map data with no relation to the trajectory of a vehicle by utilizing the RPS, so the map size used for map-matching was reduced. The data from the low-cost GPS and high-end GPS/INS device was updated once per second, and all data was transferred to the laptop. The proposed method was executed on the laptop in real time and updated the position without any time delay. The average running time for one single point according to the RPS were listed in Table 3. The row labeled ‘w/o RPS’ and ‘w/ RPS’ represent the average process time of the proposed method for each point, taking into account the RPS or not, respectively. It took approximately 0.066 s on a single point without utilizing the RPS, while 0.043 s with the RPS for Course 2. As the map size decreased, a faster average running time of the proposed method was achieved.

**Figure 15:**
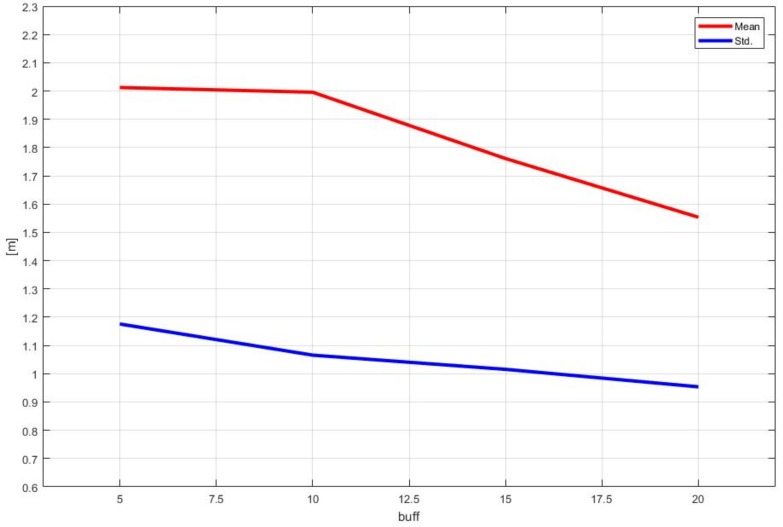
Mean and standard deviations according to the size of *buff*.

We mentioned that the performance of the proposed method is mainly dependent on the size of *buff*. Varying *buff* from 5 to 20 in Step 5, we measured the average and the standard deviation of the root mean square errors for Course 2. The results in Table 4 indicated that the proposed method provided a better performance as the size of *buff* increased, and the graphical representation of this result is shown in Figure 15. However, the increased size of *buff* increased the computational burden of operating the proposed method; thus, *buff* should be chosen according to the required accuracy and processor performance.

## 5. Discussion

The proposed algorithm assumes that a vehicle is traveling along the roads, which enables us to minimize the horizontal error, but does not correct the vertical error, as can be seen from the results. Minimizing the horizontal error is important because one can clearly determine which side of the road a vehicle is actually traveling on. Thus, the proposed method provides an advantage in the fields of car navigation and autonomous vehicle guidance.

A number of methods providing sub-meter accuracy have recently been studied. However, these methods require additional sensors or complicated algorithms, which increase the implementation cost. Since the proposed method uses only a low-cost GPS receiver and map data, the computational efficiency can be enhanced in real time. In addition, more accurate trajectories are anticipated to be obtained by applying additional sensor fusion algorithms to the trajectory corrected by the proposed method.

## 6. Conclusions

In this paper, we proposed a method for improving the position accuracy of a low-cost GPS receiver for car navigation systems using pre-built map data. In our method, the position and the direction of the points extracted from the roads were utilized as the map data. The ICP method was employed to match the points between the roads and the vehicle trajectory. Moreover, only those roads parallel to the vehicle trajectory were used rather than the entire roads. We also used the least squares method to correct the error caused by the rotation of the ICP algorithm and linked on the digital map to eliminate the residual disparity. Finally, we performed the proposed method in real time with a low-cost embedded system. Consequently, we verified that the proposed method improved the position accuracy of the low-cost GPS in real time.

## Figures and Tables

**Table 1:** Length and number of GPS points of the experimental courses.

	Course 1	Course 2	Course 3	Course 4	Course 5	Course 6
Length (km)	1.46	1.82	1.85	5.55	2.15	1.67
Number of GPS points	220	249	212	647	281	191

**Table 2:** Comparison results.

Course	Avg. (GPS) (m)	Std. (GPS) (m)	Avg. (Prop.) (m)	Std. (Prop.) (m)	Improv. (%)
Course 1	3.6013	1.7243	2.0023	1.1261	44.4006
Course 2	4.1199	1.3088	1.5535	0.9537	62.2927
Course 3	3.7037	1.1829	1.9854	1.0841	46.3941
Course 4	3.7408	2.1175	2.0153	2.2406	46.1264
Course 5	2.2805	0.8799	1.5031	0.6587	34.0890
Course 6	3.6874	1.5508	1.4432	1.2074	60.8613

**Table 3:** The average running time for a one single point according to the RPS.

	Course 1	Course 2	Course 3	Course 4	Course 5	Course 6
w/o RPS (s)	0.059	0.066	0.056	0.178	0.071	0.049
w/ RPS (s)	0.036	0.043	0.034	0.119	0.046	0.031

**Table 4:** Mean and standard deviations of Course 2 according to the size of *buff*.

*buff*	5	10	15	20
Mean (m)	2.0123	1.9957	1.7611	1.5535
Std. (m)	1.1759	1.0657	1.0155	0.9537
